# Employ of Citrus By-product as Fat Replacer Ingredient for Bakery Confectionery Products

**DOI:** 10.3389/fnut.2020.00046

**Published:** 2020-04-16

**Authors:** Cinzia Caggia, Rosa Palmeri, Nunziatina Russo, Rosario Timpone, Cinzia L. Randazzo, Aldo Todaro, Salvatore Barbagallo

**Affiliations:** ^1^Di3A – Dipartimento di Agricoltura, Alimentazione e Ambiente, University of Catania, Catania, Italy; ^2^Citrech, Viale Regina Margherita, Messina, Italy; ^3^SAAF - Dipartimento di Scienze Agrarie, Alimentari e Forestali, University of Palermo, Palermo, Italy

**Keywords:** orange juice waste, bakery confectionery products, functional foods, dietary fiber, fat replacement

## Abstract

Citrus fruits processing is one of the foremost industrial activities in Sicily and the main residual by-product consists in peels and seeds (known as “pastazzo”). Traditionally this by-product has been used for different purposes, and only most recently, it has been described as source of a wide range of healthy bioactive compounds and dietary fibers. In the present work, a debittered food grade orange fiber (DOF), extracted from orange juice by-product, was experimentally obtained and tested as fat-replacer at different percentages (30, 50, and 70%) in bakery confectionery products (brioches). The DOF showed high total fiber content, low water activity and a high water binding capacity. The obtained bakery products were characterized for nutritional, technological and microbiological parameters through storage at room temperature. Results highlighted that the addition of DOF results in final products with increased moisture content, mainly after 1 day of storage, and good textural proprieties. Furthermore, the fat-replacing strategy, at different levels of DOF, resulted in final products with, besides a constant content of carbohydrates, showed lowered fat content, increased content of dietary fiber and protein. In particular, the 50% fat replacement allowed to obtain brioches with improved technological properties and with desirable microbiological traits, mostly within the first 24 h from production and up to 5 days of storage.

## Introduction

In recent years, consumers' interest for healthy food has addressed the industry to review nutrient quality of product portfolios in order to offer products with reduced fat content (mainly saturated ones), sugar and salt added, through the addition of functional ingredients and the improvement of technological characteristics (1, 2). Several studies have showed that the adding of ingredients of plant origin allows the increase of nutritional value of final products and, generally, provides a health benefit, for their antioxidant content and proteins of high biological value (3, 4). Furthermore, food-products fortified by natural ingredients are greatly acceptable by consumers, especially by those more sensitive to environmental matter. Vegetable waste and vegetable by-products could be converted into edible ingredients and therefore should be regarded as a source of valuable components (5).

Citrus is one of the main fruit crops in the world, with an estimated production higher than 170 million tons, among which the 58% is represented by oranges (6). Italy is, after Spain, the main citrus producing country in the Mediterranean basin, and the two countries covered the 80% of the European production [USDA (7)]. In Sicily, an average of 34% of citrus fruits are processed into juices providing about a half of its weight as waste (8), which reaches 24.3 million tons per year (9). Citrus waste is mainly constituted by peels (albedo and flavedo), seeds, fruit pulp and essential oils (10) and its disposal represents an environmental concern. In this contest, several efforts have been done in different fields, suggesting citrus waste as fertilizer, feed ingredient, source for several compounds extraction (11) mainly soluble sugars, organic acids, amino acids, proteins, minerals, oils, lipids, vitamins, essential oils, pectin, or matrix for bio oil, charcoal production, and heavy metals decontaminant (12). More recently, citrus waste has been recognized as a potential source of bioactive compounds, such as flavonoids (13, 14) and dietary fiber (15, 16). Based on water solubility, fibers are distinguished into soluble (oligosaccharides, pectins, β-glucans, and galactomannan gums alginate, psyllium) and insoluble (cellulose, hemicellulose, and lignin) (17–19). Insoluble fiber seems to play an important role in the prevention and treatment of obesity, atherosclerosis, coronary heart diseases, diabetes, hemorrhoids, hypercholesterolemia, diverticular disease and colon cancer (20). Moreover, the majority of insoluble fiber is fermented in the large intestine, supporting the growth of beneficial intestinal microbiota. Its employment, alone or in association with other micronutrients, such as omega-3 fatty acids, phytosterol and probiotics (21, 22), affects functional and technological traits of several kinds of food, such as meat products (11), bread, biscuits, cookies, ice-cream (23) soft drinks and pasta (5, 24, 25). Furthermore, fortifying bakery products by adding ingredients of vegetable origin, in a final amount higher than 5% (high biological value), has encouraged several studies on products containing different functional ingredients.

Bakery confectionery products (as brioches) are complex foods, containing, beyond the main ingredients (flour and water), other optional ingredients such as salt, sugar, leaven, lipids, eggs, etc., in different proportions. The used ingredients make brioches highly caloric, bringing to diet significant amounts of complex carbohydrates, sugars and fats. However, each added ingredient, according to a particular sequence and in combination with subsequent technological steps, contributes to texture and shelf life of final products.

The aim of the present study was to produce a debittered orange fiber (DOF) powder and to be employed as fat replacer in the production of fiber fortified bakery confectionery products (brioches). Physico-chemical, nutritional and technological characteristics of obtained samples were determined and shelf life was evaluated up to 5 days.

## Materials and Methods

### Production of Debittered Orange Fiber

The debittered orange fiber (DOF) was obtained through several steps in a pilot scale process set up within the project “Uso sostenibile dei sottoprodotti provenienti dalla lavorazione industriale degli agrumi” founded by Ministero dello Sviluppo Economico (data not published). Peels from fruit processing line (“pastazzo”), were firstly crushed through hammer mills and a paddle finishers. The peels were then washed, for three times in upstream, and debittered by an alkaline solution (NaOH) at different times (30, 60, 90, and 120 min) to remove sugars, flavonoids and limonoids. After removing bitter liquids from peels, they were neutralized by a citric acid solution and finally raw pressed and dried using a fluid-bed dryer. Dried peels were coarsely milled by an hammer mill and reduced in powder with a mill up to a final mesh of 50 μm.

The waste waters were evaluated for content in limonin, according to 26). The main characteristics of the obtained insoluble DOF are shown in Table 1.

**Table 1:** Physico-chemical characteristics and composition of DOF.

**Parameter**	**Value**
Moisture	7.85 ± 0.02 g/100 g
Ash	5.67 ± 0.15 g/100 g
Dietary fiber	70.5 ± 1.0 g/100 g^*^
Pectin	1.60 ± 0.10 g/100 g^*^
Pectin ossalate soluble	0.65 ± 0.02 g/100 g^*^
Pectin water soluble	0.90 ± 0.02 g/100 g
Water binding capacity	800 ± 40%
Activity water (Aw)	0.24 ± 0.01
Limonin	<2 mg/kg of fiber
Total polyphenols	<100 mg/kg^**^

* expressed as galacturonic acid;

** *expressed as esperidin*.

### Characterization of Debittered Orange Fiber

The DOF were tested for weight, moisture and activity water (Aw). The weight was evaluated by an analytical balance (Gibertini). Moisture content was determined by automatic moisture analyzer (Gibertini) at 110°C, while, Aw was determined by AquaSorp Isotherm Generator (Decagon) at 25°C. Analyses were carried out in triplicate at T0, T1, and T5 sampling times. The total phenolic content (TPC) of DOF was determined according to Singleton and Rossi (26) method, using the Folin–Ciocalteau reagent (FC). An aliquot of the extract (250 μL) was mixed with the FC reagent (1.25 mL) and allowed to react for 3 min, then 2.5 mL of 2% sodium carbonate (Na_2_CO_3_) was added. The mixture volume was adjusted to 25 mL with distilled water and allowed to stand in the dark for 1 h. The absorbance was measured at 725 nm (Perkin Elmer lambda 25 UV-VIS spectrometer) and results were expressed as mass of gallic acid equivalents (mg/g fresh weight).

### Brioches Preparation

Brioches, common bakery confectionary products, were prepared according to traditional methods in an artisan confectionary, located in Acireale, south Italy. The recipe used for conventional brioches (here considered as control: CTR), based on 1 kg wheat flour, provided for fat (220 g), sugar (250 g), leaven (20 g), eggs (5), and salt. Experimental brioches (EB) were obtained replacing fat content, at different percentages (30, 50 and 70%) with DOF. Samples were produced between March and July 2018, and stored in sealed bags at room temperature. Physico-chemical and nutritional characteristics were determined starting from the same day of production (T0), after 1 day (T1) and after 5 days (T5).

### Determination of Physico-Chemical Parameters

Samples of CTR and EB, obtained with different fat percentage replacements, were tested for weight, moisture and activity water (Aw).

The weight was evaluated by an analytical balance (Gibertini). Moisture content was determined by automatic moisture analyzer (Gibertini) at 110°C, while, Aw was determined by AquaSorp Isotherm Generator (Decagon) at 25°C. Analyses were carried out in triplicate at T0, T1, and T5 sampling times.

### Proximate Composition of Debittered Orange Fiber

The total dietary fiber was determined in accordance with AOAC 991.43 method, using the enzymatic assay kit Total Dietary Fiber (Megazyme International Ireland Ltd, Wiclkow), following the manufacturer's instructions.

#### Determination of Fat Content

Fat content was determined according to Official Method (27). Briefly, two grams of each sample were grinded and hydrolyzed using chloridric acid. Fats were extracted with a mixture of equal volumes of ethyl ether and petroleum ether and subsequently weighed after removal of the solvent. The extraction was repeated three times, at the end of which the solvent was removed by evaporation to constant weight of the sample.

#### Determination of Protein Content

Protein content (Nx5.7) was assessed according to the Official Method (27), by a semiautomatic Kjeldal apparatus (Velp UDK139). Briefly, two grams of grinded sample were mixed with 20 ml of sulphoric acid (98%), mineralized at 420°C for 60 min, two Kjeldahl tablets of 5 g were used as catalyzer agent. The digested sample was diluted with 100 ml of distilled water, transferred in the distillation unit added with 50 ml of sodium hydroxide (0.5 M) and distilled with boric acid (4%). Finally, the distilled sample was titrated with hydrochloric acid (0.25 M).

#### Determination of Carbohydrate, Starch, and Glucose Content

The content of sugar moiety of carbohydrates was determined in accordance with the Lane and Eynon method, with some modification, as reported by Khan (28). The starch was calculated as difference.

#### Determination of Ash Content

Ash content was determined using the dry ashing technique with a muffle, in accordance to A.O.A.C (2000) method. Ten grams of grinded sample were weighed and introduced in the muffle apparatus at 550°C. The ash content was expressed on dry basis and calculated using the following equation:

$$\begin{array}{l} \text{\%Ash}\left(\text{dry basis}\right)=\left(\text{M ash}/\text{M dry}\right)\text{x }100 \end{array}$$

### Textural Properties

Textural properties were evaluated according to Maktouf et al. (29) with some modifications. The analysis was conducted by Texture Analyser for foods (Zwick Roell). Samples were compressed with a plate probe using the same speed as the firmness measurement to 60% of strain, held for 30 s, and then removed. The parameter recorded were: hardness, expressed as the peak force on first compression (Newton [N]); springiness expressed as distance of the sample recovered after the first and the second compression (mm); the maximum compression force, was determined in both experimental and control samples.

### Microbiological Analyses

An aliquot of DOF, and samples of experimental and control brioches were subjected to microbiological analyses. In detail, 1 g of DOF was diluted in sterile physiological water (0.9% NaCl) added with dimethyl sulfoxide (5.0% w/v). Serial diluted aliquots were inoculated into plates containing specific media and incubated at specific conditions, for counting different microbial groups. In details, Plate Count Agar (Sigma, Milan, Italy), incubated at 30°C for 72 h was used for mesophilic aerobic bacteria enumeration; Violet Red Bile Glucose Agar (Difco, Italy), aerobically incubated at 37°C for 24 h, for Enterobacteriaceae; Mannitol Salt Agar, incubated at 37°C for 24–48 h, for staphylococci; Sabouraud Dextrose Agar, incubated at 25°C for 72 h, for yeasts and mold and; Mc Conkey Agar (Liofilchem) incubated at 36°C for 24–28 h for *Escherichia coli* count.

Twenty-five grams of experimental and control brioches, collected at T0, T1, and T5 storage time, were diluted into sterile peptone-saline solution (225 ml) and homogenized for 3 min in a Stomacher Lab Blender 400 (International PBI S.p.A Milan, Italy). Ten-fold dilutions were obtained and aliquots (0.1 ml) were used for counting of total mesophilic bacteria and yeast and mold. Moreover, the detection of *Bacillus cereus* was carried out, according to International Standard specifies (30).

### Statistical Analyses

Rheological characteristics were determined in triplicate and separately analyzed by using the Statistical package software Minitab™ version 16.0. One-way analysis of variance (ANOVA) was performed on mean values and Fishers's test was carried out for the comparison of differences with an individual confidence interval of 95% and a simultaneous confidence level of 82.43%. Differences between sample means were considered significant at *p* ≤ 0.05.

Microbiological analyses were replicated twice for each experimental sample and sampling time, throughout storage. Microbial counts were expressed as log cfu/g and the results were reported as mean values ± standard deviation. Data were subjected to analysis of variance (ANOVA) using the XLSTAT statistical software. *p* ≤ 0.05 was considered statistically significant.

## Results

### Physico-Chemical Parameters

**Figure 1:**
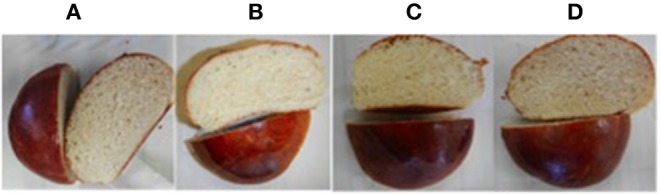
**(A)** Brioches control; **(B)** Brioches added with 30% of DOF; **(C)** Brioches added with 50% of DOF; **(D)** Brioches added with 70% of DOF.

A picture of control and experimental samples, obtained with different level of fat replacement, by DOF adding, right after the production, is shown in Figure 1.

**Figure 2:**
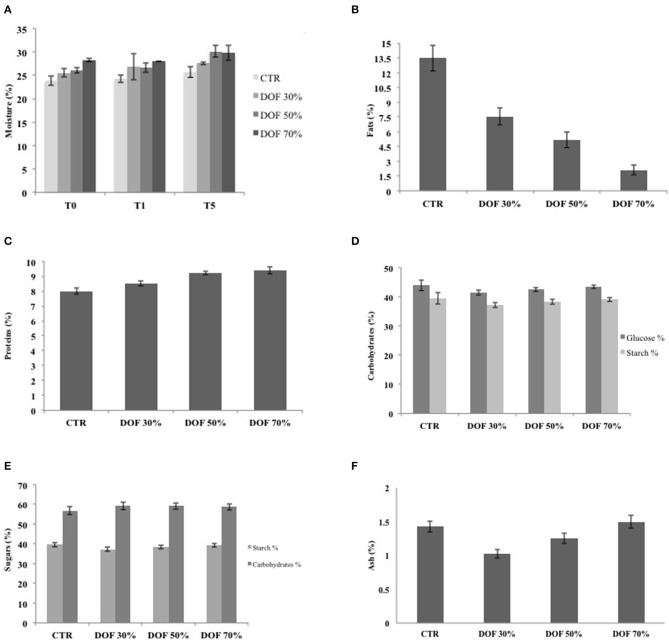
**(A)** Moisture content at different times of storage in brioches control and added with DOF; **(B)** Fats content in brioches control and added with different quantity of DOF; **(C)** Proteins content in control brioches and with different quantity of DOF; **(D)** Glucose and starch content in control brioches and with different quantity of DOF; **(E)** Carbohydrates and starch content in control brioches and with different quantity of DOF; **(F)** Ash content in control brioches and with different quantity of DOF.

Results of moisture determinations at different sampling times (T0, T1, and T5) of both experimental and control samples, stored at room temperature, are reported in Figure 2A. Data showed that experimental samples, obtained at different levels of fat replacement, showed higher moisture content than control samples, mostly after 5 days of storage (Figure 2A). These findings are in accordance to those related to weight losses (Table 2), which showed that experimental samples exhibited lower weight loss, compared to control, with DOF70% showing the lowest value, followed by DOF 30%.

**Table 2:** Changes of weight during storage and relative weight losses.

**Sample**	**T0**	**T1**	**T5**	**Weight loss (%)**
CTR	103.21^a^±4.48	99.15^a^±3.04	98.63^a^±4.69	4.43
DOF 30%	118.92^b^±3.19	116.79^b^±0.35	115.43^b^±1.38	2.93
DOF 50%	113.92^b^±2.47	112.57^b^±4.26	109.96^b^±4.33	3.47
DOF 70%	116.96^b^±0.06	114.79^b^±0.35	114.90^b^±5.68	1.76

a, b *Mean values with different letter in superscript within columns indicates significant differences (p ≤ 0.05) due to different percentage of DOF*.

Data of Aw, detected in samples during storage, did not show any significant variations among experimental samples: DOF 30% from 0.923 ± 0.010 (T0) to 0.905 ± 0.004 (T5); DOF 50% from 0.917 ± 0.007 (T0) to 0.905 ± 0.00 (T5); DOF 70% from 0.914 ± 0.005 (T0) to 0.904 ± 0.003 (T5). Whereas, a slight decrease in the control samples from 0.916 ± 0.007 (T0) to 0.899 ± 0.013 (T5), mostly after 5 days of storage, was observed.

### Nutritional Properties

Figure 2B shows the fat content of the different samples, expressed as percentage. Findings confirmed the highest fat content (10%) of control samples and a decreasing of values in experimental samples obtained with increasing DOF adding. The value dropped to 2% in samples containing 70% of fiber.

Figure 2C shows that protein contents, detected in control samples resulted lower than in experimental samples, where protein content was proportionally related to the increase of added DOF.

Regarding starch and glucose contents, as shown in Figure 2D, a variation between the starch and glucose contents in experimental samples with lower added fiber, compared to control, was detected. In particular, samples added with 30 and 50% of DOF showed a lower content of both starch and glucose. Whereas, as shown in Figure 2E, no significant variations, between experimental and control samples, were observed both for total carbohydrate and starch content. Similar results were observed for ash content (Figure 2F).

In Table 3 are reported the nutritional parameters of both experimental and control brioche samples produced with different adding of DOF.

**Table 3:** Nutritient composition of brioches.

**Parameter**	**DOF 30%**	**DOF 50%**	**DOF 70%**	**CTR**
Total dietary fiber (%)	3.99	6.65	9.31	–
Fat (%)	6.00	4.50	2.0	10.00
Protein (%)	8.50	9.20	9.40	8.00
Carbohydrates (%)	58.98	58.97	58.54	56.69
of which sugars (%)	21.85	20.7	19.46	17.2
of which starch (%)	37.13	38.27	39.08	39.49
Ash (%)	1.02	1.25	1.50	1.43

### Textural Properties

The texture of DOF-added samples was evaluated during storage at room temperature. Compression tests showed that the experimental samples showed a more homogeneous resistance to breakage when compared to the control, during the whole storage period.

Table 4 shows that the softness of control samples, after the first day of storage, was lower than those found for all experimental samples, regardless the percentage of replacement of fat. Samples containing both 50 and 70% of DOF showed significant difference after 1 day from production, but not after 5 days of storage.

**Table 4:** Cyclic compression test for brioches during storage time.

**Samples**	**T0**			**T1**			**T5**		
**Cycles**	**I**	**II**	**III**	**I**	**II**	**III**	**I**	**II**	**III**
DOF 30%	17.6^d^±0.85	18.5^d^±1.13	18.7^d^±1.13	26.55^d^±1.77	27.7^d^±1.41	29.15^d^±0.07	34.5^c^±0.99	38.85^c^±0.64	40.75^c^±3.18
DOF 50%	31.55^b^±0.64	33.05^b^±1.48	33.95^b^±1.34	37.35^c^±2.90	40.75^c^±2.47	43.01^c^±3.82	60.15^b^±6.43	58.75^b^±0.35	64.85^b^±1.48
DOF 70%	25.2^c^±3.96	26.25^c^±5.02	25.7^c^±4.53	43.45^b^±1.91	46.45^b^±4.31	47.7^b^±5.8	59.8^b^±1.98	69.55^b^±6.29	86.05^a^±5.52
CTR	46.0^a^±4.81	52.0^a^±8.06	54.2^a^±6.36	50.95^a^±0.49	85.5^a^±1.27	79.25^a^±1.2	66.3^a^±0.57	85.5^a^±1.13	104.5^a^±2.12

*The results are expressed as F (N) and compressed to 50% strain*.

a−d *Mean that in the same column followed by different superscript letters differs significantly (p < 0.05); Fisher 95% individual confidence interval, simultaneous confidence level 82.43%*.

### Microbiological Results

Results of microbiological analyses carried out on DOF samples revealed the absence of all researched microbial groups, with the exception of total mesophilic bacteria, which was recorded at a value of 4.70 log cfu/g (data non shown).

Microbiological analyses of experimental and control brioche samples showed that all microbial groups, at T0 were present at densities lower than the detection limit. As reported in Table 5, at T1 sampling the total mesophilic bacteria was recorded at an average value of 3.0 log cfu/g in all samples, except in samples obtained with 50% DOF, for which all microbial groups were below the detection limit (Table 5). Regarding yeast and mold, the lowest density (2.3 log cfu/g) was achieved in control samples while values of 2.71 and 3.02 log cfu/g were counted in experimental samples with 70% and 30% of DOF, respectively. Regarding *B. cereus* a density of about 1 log cfu/g was observed in all samples.

**Table 5:** Microbial counts and standard deviation detected in experimental and control samples during storage.

	**T1**			**T5**		
	**Total mesophilic bacteria**	**Yeast and mould**	***B. cereus***	**Total mesophilic bacteria**	**Yeast and mould**	***B. cereus***
CTR	3.02 ± 0.03^b^	2.30 ± 0.00^b^	1.00 ± 0.02^b^	6.36 ± 0.00^c^	6.85 ± 0.01^c^	1.84 ± 0.02
DOF 30%	3.02 ± 0.03^b^	3.02 ± 0.03^c^	1.02 ± 0.01^b^	4.95 ± 0.01^b^	4.91 ± 0.01^b^	1.86 ± 0.02
DOF 50%	<1^a^	<1^a^	<1^a^	4.86 ± 0.02^b^	4.95 ± 0.01^b^	1.82 ± 0.01
DOF 70%	3.09 ± 0.02^b^	2.71 ± 0.02^c^	1.01 ± 0.02^b^	4.54 ± 0.09^a^	4.59 ± 0.02^a^	1.87 ± 0.02

a−c *Mean values with different letter in superscript within columns indicates significant differences (p ≤ 0.05) due to different percentage of DOF*.

It is interesting to point out that after 5 days of storage significant differences were observed among control and experimental samples for all microbial groups, except for *B. cereus*, with an average increase of 3.4- and 1.6-log unit for yeast and mould and total mesophilic bacteria, respectively. On the contrary, no significant difference was detected in microbial counts in experimental samples obtained with 30 and 50% of added DOF (Table 5).

## Discussion

The addition of dietary fiber to bakery products represent a promising strategy to improve their nutritional quality. However high variability in fiber composition, functional and microbiological proprieties has been observed and mostly related to the applied extraction treatments. Extrusion-cooking, canning, grinding, boiling, can alter physico-chemical properties of fiber and, in some cases, improving its functionality. Lario et al. (10) demonstrated that dietary fiber opportunely obtained by lemon waste showed good functional, microbiological, and physico-chemical characteristics. Larrauri (31) showed that fiber content fiber of powder obtained from mango peels was affected by the particle size and time of peels washing, while Larrea et al. (32) reported that the extrusion process positively modified functional and structural properties of fiber obtained from orange pulp.

In the present work, a debittered orange fiber (DOF) was extracted from orange juice by-products, and produced in a pilot scale. The technique applied for DOF production reduced the limonoids content that contributes to bitterness of fiber, strongly compromising its usability (33, 34). In addition the final spry-drying, allowed obtaining a product with a low Aw value (0.24) that inhibits the microbial growth, making the DOF a microbiological stable and a safe product. The presence of mesophilic aerobic bacteria, in this case, represents an indicator of hygiene process and could be related to packages and/or to erroneous manipulation in post processing phases. The European Food Safety Authority (35) reported that the different physico-chemical characteristics of a fiber are not only related to its fermentability and other physiological effects, but mainly to bulking property due to the water holding capacity (36). The DOF here obtained showed good functional properties with a high water and oil holding capacities, which are the most important functional parameter for the employ of fiber in bakery product (37).

Brioches are semi-preserved foods, and their stability depends on a variety of factors such as moisture content, process, storage temperature and presence of chemical additives or stabilizers. In the present study different levels of DOF were used as fat replacer in brioches and the effects on physico-chemical, technological, nutritional, and microbiological properties were evaluated at different time of storage at room temperature. The addition of fibers, besides improving nutritional characteristics, leads an overall improvement, mainly from a technological point of view, according to Martins et al. (38). In particular, the replacement of fat with DOF produced positive effects, arising brioches with higher moisture contents, higher protein and similar activity water and carbohydrates values than controls, confirming that fat replacement with DOF is a strategy suitable firstly to increase fiber and secondly to reduce fat contents (39, 40). In bakery confectionary products, carbohydrate, starch and glucose are of great importance, representing the main constituent that can give rise to qualitative changes during storage. The addition of DOF do not influence the content in both starch and glucose, moreover no variation was observed in total carbohydrate content. The experimental samples showed, also, improved rheological properties, Fmax values and their differences are lower respect to the control, as confirmed by compression test, and good microbiological characteristics, mainly within the first day from production, which is the most frequent time of consumption. Moreover, although lipids represent secondary ingredients, they perform important technological functions in doughs, such as aeration, gas retention in glutinous mesh, plasticizing action, anti-stress action, improvement of sensory characteristics and lubricating action, their replacement with DOF did not affect rheological proprieties of final products. Here the replacement of fat, with different percentages of DOF, resulted in a decrease of fat up to 2%, in samples containing 70% of fiber. Crizel et al. (41) evaluated the application of fibers originated from orange waste as a fat replacer in ice cream obtained final products with a 70% reduction of fat, without any significant changes in color, odor and texture. The incorporation of orange fiber by-products in fresh pasta was also recently considered and results showed an increase of both antioxidant capacity and fiber content in final product, without any detrimental effect (24).

In the present study, DOF was incorporated at higher amount than in previous studies (42, 43) and experimental brioches showed higher content of protein, and lower percentage of ash (at 30 and 50% of fat replacement) in discordance to findings reported by Nassar et al. (40).

For brioches, as for other bakery confectionary products, with high or intermediate moisture content, microbial spoilage is often the major factor limiting the shelf life and a major cause of economic loss (44). The artisanal brioches are supposed to be consumed as fresh products and are daily produced for direct sales, without any packages. For this reason, they are mainly susceptible to post baking contamination, mainly by mould, that are microrganisms well-adapted to xerophilic conditions (45). The contamination can occur by bakery surfaces and equipment, by food handlers, and, above all, by raw ingredients (44). Mould spoilage are more troublesome during the summer months, due to the warmer and more humid storage conditions. A major source of *Bacillus* contamination is represented by raw ingredients (e.g., flour, sugar, and leaven), and the microorganism survives the baking process, germinates upon cooling, and grows under both aerobic and anaerobic conditions. Results obtained from microbiological analyses confirmed the antimicrobial effects reported for natural extracts from fruit and vegetables by-products that may contain antimicrobial agents, and thus used as an additional control measure to guarantee the food safety (5)

The amount of fiber in the food can vary from (46) <0.2 g to 20 g/serving and the EU Regulation (EC) No 1924/2006 specifies the use of the term “source of fiber” as referred to products containing at least 3 g of fiber per 100 g and the terms “high in fiber” as referred to product containing at least 6 g of fiber per 100 g. Results of present work demonstrated that the flat replacement with DOF at 50% produced brioche with final dietary fiber content enough to be referred as brioches with high fiber content. Many experts recommend a total dietary fiber intake of 25–30 grams per day end (35), proposed that a fiber intake of 25 g/d would be adequate for normal laxation in adults whereas more than 25 g/d is required to reduce risk of coronary heart disease, type 2 diabetes and improved weight maintenance (36). Surveillance data showed that dietary fiber intakes, among adults in Italy, is estimated around 19 g/d (47) and any effort must be done in order both to promote consumption of food with high fiber content and to offer fortified products with good quality traits. Taking into account the recommended daily intake of dietary fiber, the consumption of one brioche obtained with the 50% of fat replacement, can cover from 21.6 to 25.2% and from 23.65 to 30, 28% the recommended daily amount for men and women, respectively.

## Conclusion

An increasing attention has been globally paid on the utilization of fruit processing by-products that could be converted into edible products and/or ingredients. Such a use contributes to a better utilization of important resources and has recently resulted in the appearance of commercial various new ingredients, mainly containing dietary fiber. The use of by-products is of interest, both for reducing industrial environmental contamination and increasing functional, technological and nutritional properties of food. The present study demonstrated that the addition of debittered orange fiber resulted compatible with bakery products processing enhancing stability of final products. In addition, the fat strategy replacing, improved nutritional traits of a bakery confectionary product largely consumed in Italy, leading the obtaining a low-fat brioches fortified with natural fiber.

## Data Availability Statement

The raw data supporting the conclusions of this article will be made available by the authors, without undue reservation, to any qualified researcher.

## Author Contributions

CC, CR, and SB: substantial contributions to the conception and design of the work, acquisition, analysis, and interpretation of data. RP, NR, RT, and AT: acquisition, analysis and interpretation of data. CC, RP, and CR: drafting the work and revising it critically for intellectual content. All authors approved the final version of the manuscript to be submitted for publication and agreed to be accountable for all aspects of the work in ensuring that questions related to the accuracy and integrity of any part of the work are appropriately investigated and resolved.

### Conflict of Interest

The authors declare that the research was conducted in the absence of any commercial or financial relationships that could be construed as a potential conflict of interest.
